# Clinical features of inflammatory bowel disease unclassified: a case-control study

**DOI:** 10.1186/s12876-024-03171-5

**Published:** 2024-03-13

**Authors:** Yupei Shao, Yixiao Zhao, Hong Lv, Pengguang Yan, Hong Yang, Jingnan Li, Ji Li, Jiaming Qian

**Affiliations:** 1 Department of Gastroenterology, Peking Union Medical College Hospital, Chinese Academy of Medical Science & Peking Union Medical College, No.1 Shuai Fu Yuan, Dong Cheng District, 100730 Beijing, China; 2 Department of Gastroenterology, Civil Aviation General Hospital, 100025 Beijing, China

**Keywords:** Inflammatory bowel disease, Inflammatory bowel disease unclassified, Indeterminate colitis, Clinical features, Prognosis

## Abstract

**Background:**

Approximately 10-15% of inflammatory bowel disease (IBD) patients with overlapping features of ulcerative colitis (UC) and Crohn’s disease (CD) are termed as inflammatory bowel disease unclassified (IBDU). This study aimed to describe the clinical features of IBDU and evaluate the potential associated factors of reclassification.

**Methods:**

The clinical data of 37 IBDU patients were retrospectively analyzed from November 2012 to November 2020. 74 UC and 74 CD patients were randomly selected and age- and sex-matched with the 37 IBDU patients. Clinical characteristics were compared between the three patient groups. Potential factors associated with the IBDU reclassification were evaluated.

**Results:**

60% of IBDU patients displayed rectal-sparing disease, and 70% of them displayed segmental disease. In comparison to UC and CD, the IBDU group demonstrated higher rates of gastrointestinal bleeding (32.4%), intestinal perforation (13.5%), spontaneous blood on endoscopy (51.4%), and progression (56.8%). The inflammation proceeded relatively slowly, manifesting as chronic alterations like pseudopolyps (78.4%) and haustra blunt or disappearance (56.8%). 60% of IBDU patients exhibited crypt abscess, and 16.7% of them exhibited fissuring ulcers or transmural lymphoid inflammation. The proportions of IBDU patients receiving immunosuppressants, surgery, and infliximab were basically the same as those of CD patients. During the 79 (66, 91) months of follow-up, 24.3% of IBDU patients were reclassified as UC, while 21.6% were reclassified as CD. The presence of intestinal hemorrhaging was associated with CD reclassification, while hypoalbuminemia was associated with UC reclassification.

**Conclusions:**

IBDU may evolve into UC or CD during follow-up, and hemorrhage was associated with CD reclassification. Different from the other two groups, IBDU exhibited a more acute onset and a gradual progression. When an IBD patient presents with transmural inflammation or crypt abscess but lacks transmural lymphoid aggregates or fissuring ulcers, the diagnosis of IBDU should be considered.

## Background

Approximately 10-15% of inflammatory bowel disease (IBD) cases cannot be accurately diagnosed as ulcerative colitis (UC) or Crohn’s disease (CD) [1, 2]. Unclassified patients with overlapping features of CD and UC are termed “inflammatory bowel disease unclassified” (IBDU). In 1970, the term “indeterminate colitis” (IC) was first proposed by Kent et al. among colectomies presenting with “overlapping features and/or insufficient evidence to make a final diagnosis” [3]. In 2005, the World Congress of Gastroenterology proposed the IBDU term for presurgical cases in which clinical manifestations, endoscopic and biopsy examinations only indicate IBD but where a further diagnosis was not possible [4].

The current incidence of IBDU varies from 1 to 20% due to variations in clinical manifestations and the lack of uniform diagnostic criteria [5, 6]. The most common causes of an IBDU diagnosis include IBD in the fulminant or early phase, insufficient clinical or pathological information, failure to identify unusual pathological variants of UC or CD, and inability to discriminate non-IBD and other comorbidities. Some experts consider IBDU a temporary diagnosis since many IBDU patients are reclassified as having UC or CD during follow-up [7], while others believe IBDU should be regarded as a third subcategory of IBD as some patients retain the diagnosis even after long-term follow-up [8]. So far, there are still ongoing arguments regarding whether IBDU represents a third subcategory of IBD or not [9].

Due to differences in optimal treatments and prognosis within IBD, early diagnosis and reclassification of IBDU is essential. It has been reported that IBDU patients with ileal pouch-anal anastomosis (IPAA) exhibited a higher prevalence of complications like pouch fistula, pelvic sepsis, and perineal diseases than UC [10]. However, there is a paucity of data on Chinese patients with IBDU, and studies regarding potential factors of IBDU reclassification are scarce. Therefore, this retrospective study aimed to: (1) compare the clinical characteristics of patients with IBDU to those with CD and UC to improve early identification; (2) evaluate the factors of UC or CD reclassification and optimize therapeutic strategies.

## Methods

### Study population

This single-center retrospective study was conducted at Peking Union Medical College Hospital (PUMCH) in China from November 2012 to November 2020. The hospitalization records of IBDU patients were assessed using the electronic medical records of PUMCH, in which the first diagnosis was IBDU or IC. Each IBDU patient was then randomly gender- and age-of-admission-matched with 2 UC and 2 CD patients from the PUMCH IBD database, and updated during the follow-up. A diagnosis of IBDU was considered when patients with IBD were unclassifiable to UC or CD after evaluating clinical manifestations, endoscopic and pathological examinations.^[4]^ This study then diagnosed IBDU by identifying one or a combination of the following indicators: segmental lesions or rectal sparing, reverse gradient of mucosal inflammation (proximal > distal), inflammation in the small bowel (confirmed through radiology or endoscopy), colonic stricture, perianal disease, deep colonic ulcers, and the presence of transmural inflammation in pathology. Each case was confirmed after the discussion by at least two gastroenterologists and pathologists. The diagnostic criteria, distribution, severity, clinical phenotype, and efficacy determination of UC and CD were all satisfied using the third European evidence-based consensus on the diagnosis and management of IBD [2, 11]. The diagnosis of all UC and CD patients was confirmed during clinical follow-up. All IBD patients had small bowel imaging (Computed Tomography Enterography or Magnetic Resonance Enterography or intestinal ultrasound) and ileocolonoscopy. All CD patients and 73% of IBDU patients had upper gastrointestinal (UGI) endoscopy screening. The exclusion criteria were: patients younger than 18 years old at admission; patients concurrent with other diseases, including infectious diseases, intestinal tuberculosis, lymphoma, severe cardiovascular disease or hepatic and renal disorders, and other gastrointestinal tumors; patients with follow-up time less than 6 months; patients with incomplete clinical data or ambiguous diagnosis.

### Follow-up observations and outcomes

All patients included in the study were continuously followed until their latest medical record of PUMCH or until November 2020, and the median follow-up time was 79 (66, 91) months. The primary outcome of IBDU was defined as reclassification as either CD or UC. Re-diagnosis was mainly based on endoscopic evaluation and pathological findings.

### Data collection

We collected patient data regarding demographic characteristics, clinical manifestations, disease extent, laboratory findings, endoscopic features, histopathology, and initial treatment at the time of diagnosis. Additionally, the following information was also collected: the progression of disease extent as seen on endoscopy, treatment, and disease reclassification during follow-up. Family history was defined as immediate or extended family members diagnosed with IBD. Complications like intestinal obstruction and perforations not only had relevant clinical symptoms, but also were confirmed by imaging examinations. Gastrointestinal hemorrhage was defined as a decrease of at least 2 g/dL in hemoglobin or the need for blood transfusion support. Medication history included 5-Aminosalicylic Acid ([5-ASA] oral, enema, or suppository), systemic glucocorticoids, immunomodulators ([azathioprine, methotrexate, and thalidomide), and infliximab (IFX). IBD-related surgery only included resection of the small bowel, segmental colectomy, and total proctocolectomy.

### Statistical analysis

Continuous variables conforming to the normal distribution were expressed as the mean ± standard deviation (sd), and the two groups were compared using the two-sample t-test. Continuous variables without a normal distribution were expressed as the median and interquartile range (IQR) were compared using the Wilcoxon rank sum test. Categorical variables are expressed in percentages (or proportions) and the data were compared using the Chi-squared test or Fisher’s exact test. Univariate COX regression analysis was conducted to investigate factors potentially associated with the UC or CD reclassification during follow-up. Parameters with a *P* value less than 0.1 in univariate analyses were introduced into multivariable COX regression with backward selection. Statistical analyses were performed using SPSS 17.0 software (SPSS Inc., Chicago, IL, USA) and a *P* value < 0.05 was considered statistically significant.

## Results

### Demographic characteristics

There were 37 patients in the IBDU group and 74 patients in the CD and UC group. The proportion of males was 64% in all three groups. The median (IQR) age at onset of IBDU, UC, and CD was 27 (21,39) years, 27 (21,39) years, and 26.5 (20,38) years, respectively. The median (IQR) duration from symptom onset to diagnosis of IBDU was 24 (11,65) months, which was shorter than the duration from symptom onset to diagnosis of CD (*P* < 0.05) [Table 1].

**Table 1:** Comparison of demographic characteristics between IBDU, UC and CD groups at diagnosis

	IBDU (37)	UC (74)	*p*-value	CD (74)	*p*-value
age at symptom onset (years), median (IQR)	27(21,39)	27(21,39)	0.846	26.5(20,38)	0.367
duration from symptom onset to diagnosis (months), median (IQR)	24(11,65)	32(14,69)	0.390	55(21,98)	0.019
BMI (kg/m^2^), mean ± sd	19.9 ± 3.0	19.9 ± 2.7	0.955	18.8 ± 3.2	0.092
smoking status at diagnosis (n, %)					
current smoker, n (%)	11(29.7%)	11(14.9%)	0.064	14(18.9%)	0.199
ex-smoker, n (%)	0(0.0%)	3(4.1%)	0.535	5(6.7%)	0.257
non-smoker, n (%)	26(70.3%)	60(81.0%)	0.199	55(74.3%)	0.650
familial history of IBD, n (%)	4(10.8%)	3(4.1%)	0.890	3(4.1%)	0.334

### Clinical manifestations and complications

The prevalence of mucopurulent bloody stool (64.9%) in IBDU was significantly higher than in the CD group (*P* < 0.01), and it was significantly lower than in the UC group (*P* < 0.01). The prevalence of anemia (62.2%) and fever (59.5%) in IBDU were comparable to CD, which was more common than UC (*P* < 0.05). Gastrointestinal bleeding was the most common complication in IBDU patients, and the incidence of gastrointestinal bleeding (32.4%) and intestinal perforation (13.5%) were higher in the IBDU group compared to the other two groups. The complications of IBDU were more similar to CD complications, including higher rates of intestinal perforation(13.5% vs. 5.4% vs. 1.4%), obstruction (32.4% vs. 48.6% vs. 4.1%), fistula (24.3% vs. 31.1% vs. 1.4%), and perianal diseases (29.7% vs. 37.8% vs. 9.5%) compared to UC patients (*P* < 0.01) [Table 2].

**Table 2:** Comparison of clinical manifestations between IBDU, UC and CD groups at diagnosis [case *n*, (%)]

	IBDU (37)	UC (74)	*p*-value	CD (74)	*p*-value
**Clinical symptoms**					
fever	22(59. 5%)	26(35.1%)	0.015	39(52.7%)	0.500
anemia	23(62.2%)	30(40.5%)	0.032	36(48.6)	0.179
abdominal pain	29(78.4%)	57(77.0%)	0.872	63(85.1%)	0.373
mucopurulent bloody stool	24(64.9%)	67(90.5%)	0.010	6(8.1%)	0.000
weight loss	27(73.0%)	53(71.6%)	0.881	53(71.6%)	0.881
**Complications**					
hemorrhage	12(32.4%)	15(20.2%)	0.089	19(25.7%)	0.300
perforation	5(13.5%)	1(1.4%)	0.009	4(5.4%)	0.128
perianal disease	11(29.7%)	7(9.5%)	0.006	31(37.8%)	0.213
fistula	9(24.3%)	1(1.4%)	0.000	32(31.1%)	0.104
obstruction	12(32.4%)	3(4.1%)	0.000	36(48.6%)	0.104

### Intestinal location

Compared with CD patients, IBDU patients had a higher prevalence of disease involvement from the transverse colon to the rectum and pancolitis (*P* < 0.01), but a lower prevalence of ileal involvement (21.6% vs. 70.3%, *P* < 0.01). The IBDU group had a higher prevalence of ileal involvement (21.6% vs. 9.5%, *P* < 0.05) and rectal sparing (59.5% vs. 2.7%, *P* < 0.01), compared with the UC group. During follow-up, IBDU patients were most likely to display a progression in intestinal involvement, with 60% of patients ending up with total colitis [Table 3].

**Table 3:** Comparison of intestinal involvement between IBDU, CD and UC groups at diagnosis and last follow-up [case *n*, (%)]

	IBDU (37)	UC (74)	*p*-value	CD (74)	*p*-value
**At diagnosis**					
terminal ileum	8(21.6%)	7(9.5%)	0.036	52(70.3%)	0.000
ileocecum	13(35.1%)	19(25.7%)	0.109	45(60.8%)	0.043
ascending colon	12(32.4%)	30(40.5%)	0.406	27(36.4%)	0.887
transverse colon	22(59.5%)	37(50.0%)	0.346	25(33.8%)	0.010
descending colon	22(59.5%)	53(71.6%)	0.197	23(31.1%)	0.004
sigmoid colon	24(64.9%)	65(87.8%)	0.004	22(29.7%)	0.000
rectum	15(40.5%)	72(97.3%)	0.000	11(14.9%)	0.000
pancolitis	8(21.6%)	28(37.8%)	0.085	9(12.1%)	0.712
**At last follow-up**					
terminal ileum	13(35.1%)	7(9.5%)	0.001	53(71.6%)	0.000
ileocecum	21(56.8%)	27(36.5%)	0.042	51(68.9%)	0.206
ascending colon	24(64.9%)	40(54.1%)	0.277	37(50.0%)	0.138
transverse colon	30(81.1%)	58(78.4%)	0.741	34(45.9%)	0.000
descending colon	28(75.7%)	64(86.5%)	0.154	31(41.9%)	0.001
sigmoid colon	30(81.1%)	70(94.6%)	0.050	25(33.8%)	0.000
rectum	22(59.4%)	74(100.0%)	0.000	12(16.2%)	0.000
pancolitis	22(59.4%)	40(54.1%)	0.833	15(20.2%)	0.000
progression	21(56.8%)	26(35.1%)	0.030	12(16.2%)	0.000

### Endoscopic findings

The prevalence of stricture (73% vs. 75.7% vs. 23%), segmental disease (70.2% vs. 70.3% vs. 10.8%) and longitudinal ulcer (40.5% vs. 63.5% vs. 16.2%) in IBDU were similar to patients with CD, and were significantly higher than UC (*P* < 0.01). The prevalence of pseudopolyps (78.4%, *P* < 0.01) and spontaneous blood (51.4%, *P* < 0.05) of IBDU was more common than the other two groups [Table 4].

**Table 4:** Comparison of endoscopic findings between IBDU, CD and UC groups at diagnosis [case *n*, (%)]

	IBDU (37)	UC (74)	*p*-value	CD (74)	*p*-value
stricture	27(73.0%)	17(23.0%)	0.000	56(75.7%)	0.757
segmental lesion	26(70.2%)	8(10.8%)	0.000	52(70.3%)	0.771
spontaneous bleeding	19(51.4%)	23(31.0%)	0.038	21(28.3%)	0.017
haustra blunt or disappearance	21(56.8%)	38(51.4%)	0.501	12(16.2%)	0.000
disordered or disappearing blood vessel texture	20(51.4%)	61(82.4%)	0.002	4(5.4%)	0.000
pseudopolyp	29(78.4%)	38(51.4%)	0.006	33(44.6%)	0.001
longitudinal ulcer	15(40.5%)	12(16.2%)	0.005	47(63.5%)	0.022

*P < 0.05, **P < 0.01

### Pathological findings

The prevalence of fissuring ulcers in IBDU was similar to UC (16.7% vs. 14.3%), and was significantly lower than CD (16.7% vs. 58.3%, *P* < 0.01). 2 IBDU patients were found with granuloma, but both of them were close to the crypt. Although the prevalence of transmural inflammation in IBDU was the same with CD (75% vs. 75%), only 2 of them (16.7%) were transmural lymphoid aggregates. The prevalence of crypt abscess in IBDU was higher than CD (60% vs. 37.5%, *P* < 0.05), but lower than UC (60% vs. 86.4%, *P* < 0.01) [Table 5].

**Table 5:** Comparison of pathological findings between IBDU, UC and CD groups at diagnosis [case *n*, (%)]

	IBDU	UC	*p*-value	CD	*p*-value
**Patients with endoscopy evaluation**	35	59		72	
atypical hyperplasia	4(11.4%)	10(16.9%)	0.467	4(5.6%)	0.489
crypt abscess	21(60.0%)	51(86.4%)	0.003	27(37.5%)	0.028
granulation tissue	19(54.3%)	26(44.1%)	0.338	56(77.8%)	0.013
granuloma	2(5.7%)	1(1.7%)	0.642	22(30.6%)	0.004
**Patients with colectomies**	12	7		24	
fissuring ulcer	2(16.7%)	1(14.3%)	1.000	14(58.3%)	0.032
submucosa lymphoid aggregate	4(33.3%)	1(14.3%)	1.000	18(75.0%)	0.029
transmural lymphoid aggregate	2(16.7%)	0(0.0%)	0.509	14(58.3%)	0.032
transmural inflammation	9(75.0%)	0(0.0%)	0.003	18(75.0%)	1.000

### Treatment

The proportion of IBDU patients treated with immunosuppressants (62.1% vs. 68.9% vs. 33.8%), surgery (40.5% vs. 36.5% vs. 10.7%) and IFX (24.3% vs. 28,4% vs. 17.6%) was similar with CD, and was higher than UC patients (*P* < 0.01), but the latter was not statistically significant. In addition to infliximab, two IBDU patients opted for adalimumab and ustekinumab, respectively, owing to insufficient response to IFX. Eventually, these two patients underwent surgical intervention due to the development of intestinal stricture and resistance to conventional medical treatments. Among UC patients, two cases with inadequate response to IFX were treated with vedolizumab. Within the CD patient cohort, five individuals switched to adalimumab, and one to ustekinumab due to inadequate response to IFX. A total of 15 IBDU patients (40.5%) underwent surgery and the median (IQR) time from disease onset to surgery was 56 (16, 101) months. IPAA surgery was given to 4 IBDU patients (26.7%) due to refractory medical treatment and gastrointestinal hemorrhage. Among IBDU patients undergoing IPAA surgery, 2 were reclassified as UC and 1 was reclassified as CD during follow-up. 3 UC patients underwent IPAA surgery, and no postoperative complications was observed in the UC and IBDU group. However, only 1 CD patient (3.7%) underwent IPAA surgery and was found with a pouch fistula after surgery. For surgical indications, becoming refractory to medical treatment was the most common reason for UC patients to require surgery, while complications like hemorrhage were the main reason for surgery in IBDU and CD patients [Table 6].

**Table 6:** Comparison of treatment between IBDU, CD and UC groups at last follow-up

	IBDU (37)	UC (74)	*p*-value	CD (74)	*p*-value
surgery, n (%)	15(40.5%)	8(10.7%)	0.000	27(36.5%)	0.678
time from disease onset to surgery (months), median (IQR)	56(16, 101)	56.5(43.75, 115.25)	0.540	21(12, 55.5)	0.222
surgery complication, n (%)	3(8.1%) anastomotic ulcer 1 anastomotic stenosis 2	0(0.0%)		6(8.1%) anastomotic ulcer 2 anastomotic fistula 1 anastomotic stenosis 3	
corticosteroid, n (%)	36(97.2%)	68(91.9%)	0.897	58(78.4%)	0.029
5-ASA, n (%)	37(100.0%)	74(100.0%)	--	67(90.5%)	0.129
IM, n (%)	23(62.1%)	25(33.8%)	0.004	51(68.9%)	0.477
IFX, n (%)	9(24.3%)	13(17.6%)	0.400	21(28.4%)	0.650
time from disease onset to the use of IFX (months), median (IQR)	80(63,116.5)	35(15,81)	0.035	69(17,113)	0.428
courses of IFX, n median (IQR)	12(7.5,17.5)	6(4,10)	0.032	12(7,15)	0.964

### The outcome of IBDU

The median follow-up of 79 (66, 91) months revealed that 8 patients (21.6%) were reclassified as definite CD from onset to 90 months later, and 9 patients (24.3%) were reclassified as definite UC from onset to 50 months later. In the 9 IBDU patients diagnosed with UC, due to the initial endoscopic presence of non-diffuse disease, multiple stenosis, rectal sparing, and the pathological presence of fissuring ulcers, these patients could not initially be definitively diagnosed. During the follow-up period, 6 patients were reexamined, and endoscopic findings were consistent with typical UC patterns, and 3 patients underwent surgical intervention with postoperative pathology features entirely in line with UC. In the 8 IBDU patients diagnosed with CD, the lack of diagnostic pathology findings of CD led to no final diagnosis initially. During follow-up, 5 patients had a postoperative pathology in line with CD. 3 patients reached the final diagnosis owning to colonoscopy findings strongly in favour of CD. In comparison to patients reclassified to UC, those still diagnosed with IBDU exhibited a higher level of serum albumin at diagnosis (*P* < 0.05). Contrasted with patients reclassified to CD, IBDU patients demonstrated a reduced incidence of hemorrhage (15.0% vs. 62.5%, *P* < 0.05) and surgery (30.0% vs. 75.0%, *P* < 0.01) [Table 7].

**Table 7:** Comparison of features between IBDU, CD-reclassified and UC-reclassified groups [cases (%)]

	IBDU (20)	UC- reclassified (9)	*p*-value	CD- reclassified (8)	*p*-value
albuminemia (g/L), mean ± sd	35.0 ± 6.2	30.4 ± 6.5	0.049	33.0 ± 6.9	0.386
mucopurulent bloody stool, n (%)	12(60.0%)	8(88.9%)	0.201	4(50.0%)	0.691
hemorrhage, n (%)	3(15.0%)	4(44.4%)	0.158	5(62.5%)	0.022
perforation, n (%)	1(5.0%)	2(22.2%)	0.220	2(25.0%)	0.188
perianal disease, n (%)	4(20.0%)	2(22.2%)	1.000	5(62.5%)	0.068
fistula, n (%)	4(20.0%)	1(11.1%)	0.654	4(50.0%)	0.172
obstruction, n (%)	6(30.0%)	1(11.1%)	0.382	5(62.5%)	0.200
rectal involvement, n (%)	7(35.0%)	5(55.6%)	0.422	3(37.5%)	1.000
stricture, n (%)	13(65.0%)	7(77.8%)	0.675	7(87.5%)	0.371
segmental lesion, n (%)	15(75.0%)	6(66.7%)	0.483	5(62.5%)	0.651
longitudinal ulcer, n (%)	8(40.0%)	2(22.2%)	0.431	5(62.5%)	0.410
crypt abscess, n (%)	11(55.0%)	5(55.6%)	1.000	5(62.5%)	1.000
granuloma, n (%)	1(5.0%)	0(0.0%)	1.000	1(12.5%)	0.497
Surgery, n (%)	6(30.0%)	3(33.3%)	1.000	6(75.0%)	0.044
Corticosteroid, n (%)	19(95.0%)	9(100.0%)	1.000	8(100.0%)	1.000
IM, n (%)	9(45.0%)	7(77.8%)	0.130	7(87.5%)	0.088
IFX, n (%)	4(20.0%)	1(11.1%)	1.000	4(50.0%)	0.172

### Associated facrors with UC or CD reclassification

Univariate Cox regression analysis showed that hypoalbuminemia (HR 0.9, 95% CI 0.82–0.99, *P* = 0.03) at diagnosis was associated with UC reclassification, while complications of hemorrhage (HR 4.8, 95% CI 2.3–20.4, *P* = 0.03) were associated with CD reclassification. Multivariate Cox model analysis demonstrated that patients with lower levels of albuminemia (HR 0.9, 95% CI 0.79–0.98, *P* = 0.023) had a higher probability of re-diagnosis as UC, while patients with hemorrhage (HR 4.2, 95% CI 2.0-17.9, *P* = 0.05) had a higher probability of re-diagnosis as CD. Although our analysis indicated that patients with complications like fistula and obstruction, longitudinal ulcer on endoscopy, granuloma, and treatment of IFX were more inclined to be reclassified as CD than UC, the differences were not statistically significant.

## Discussion

The research on the clinical course of IBDU is limited. This retrospective study mainly described the clinical features of IBDU and evaluated associated factors of IBDU reclassification during follow-up. After 79 (66, 91) months of median follow-up time, more than half of IBDU patients maintained their initial diagnosis, supporting that IBDU could be considered as a third subcategory of IBD. Compared to the two other groups, IBDU typically exhibited a more fulminant onset with a gradual progression over time. In this study, although the clinical features and treatment pattern of IBDU were more similar to CD, most of them failed to present definitive pathological markers of CD. This indicates that the identification of IBDU does not rely on a ‘positive’ diagnosis but rather on the absence of distinct diagnostic findings pointing to either CD or UC. Clinical features like hemorrhage, surgery intervention and hypoalbuminemia at diagnosis may serve as a potential factor for IBDU reclassification. For IBDU patients concurrent with hemorrhage, a more cautious attitude should be taken to IPAA surgery.

In our study, more than 50% of IBDU patients displayed rectal sparing and segmental lesions, both of which were crucial details aiding in the diagnosis of IBDU. Besides, the IBDU group exhibited higher rates of gastrointestinal bleeding, intestinal perforation, and spontaneous blood on endoscopy compared to the other two groups. This was likely because IBDU patients were commonly recognized as having fulminant colitis and an acute disease onset [12]. Severe inflammation in patients typically manifests primary involvement of the more proximal colon, and the varying levels of inflammation throughout the colon may lead to the so-called “skipping lesions” [13]. Moreover, the progression of intestinal involvement in IBDU was significantly higher than that observed in UC and CD. However, the inflammation advanced relatively slowly, featuring chronic changes such as pseudopolyps and haustra blunt or disappearance. Over time, it eventually involved all layers of the intestinal wall, resulting in complications such as intestinal fistulas and obstructions. Another review comparing symptoms of UC, CD and IBDU indicated that both clinical and endoscopic manifestations of IBDU were variable [14]. Therefore, accurate diagnosis of disease is time-dependent, and careful and repeated review of biopsy materials is essential.

On the other hand, most IBDU patients lacked diagnostic pathological findings of CD in our study. For example, although the prevalence of transmural inflammation in our IBDU group was almost the same as in the CD group, few IBDU patients were found with transmural lymphoid aggregate and granulomas. In other words, transmural inflammation seen in IBDU patients lacks the presence of transmural lymphoid aggregates, which serve as a strong pathognomonic indicator of CD [15]. Only 16.7% of IBDU patients displayed fissuring ulcers, a percentage much lower than the 58.3% observed in CD patients. Conversely, the incidence of crypt abscess (60%) was notably higher in IBDU patients compared to CD patients (37.5%). Therefore, in cases where IBD patients present with transmural inflammation or crypt abscess but lack transmural lymphoid aggregates or fissuring ulcers, we tend to make a diagnosis of IBDU. In addition, special attention needs to be paid to the identification of granulomas and fissures. The typical granuloma of CD is clearly defined and sarcoid-like transmural, but up to 30% of UC patients had epithelioid granulomas associated with denatured collagen, infection and drug reactions [16–19]. Therefore, mucosal granulomas, especially those close to inflamed and ruptured crypts, may be found in both IBDU and UC patients, which was consistent with our patients.

At present, there are few large-scale prospective studies on medical treatments of IBDU and patients are generally managed using the basic treatment principles of IBD. For IBDU patients with no response to conventional medical interventions, IFX and surgery are regarded as promising alternatives [20]. However, in comparison with CD, IBDU patients exhibited a poor therapeutic response to IFX [21]. Both adult and pediatric cohorts have revealed that the treatment patterns of IBDU are more inclined to UC, with fewer receiving early immunosuppression, biologics and surgical intervention than CD [21, 22]. In contrast, our study showed that IBDU patients had a higher risk of taking immunosuppressants, IFX and surgical intervention than UC patients. This may be attributed to variations in the length of follow-up and a higher prevalence of steroid dependence or resistance. Due to constraints related to economic factors and indications, the utilization of novel biologics such as adalimumab or ustekinumab is limited in our IBDU population. Based on the observed administration, these biologics have demonstrated suboptimal efficacy in IBDU patients. With the gradual inclusion of these biologics in medical insurance coverage, there is a need for a multi-center and prospective study to gather more comprehensive clinical data. Our future endeavors aim to explore variations in therapeutic efficacy and identify optimal indications for different biologics within the IBDU population.

Birimberg-Schwartz et al. supposed that IBDU was a distinct phenotype within the category of IBD, arguing that its prevalence in adults has remained stable at around 10% for the last three decades, even with the introduction of advanced diagnostic techniques [23]. It has been reported that as many as 80% of IBDU patients were reclassified to UC or CD or even non-IBD at the 8-year follow-up [24], but a recent European prospective study showed that 84% of IBDU patients remained unclassified after 6 months [22], which indicates that the number of UC or CD as a subsequent diagnosis increases as follow-up time increases. Among patients whose diagnosis was reclassified, the bulk of the final diagnosis was UC, while 10-30% behaved like CD [22, 25]. During our 6-year follow-up, 9 patients (25%) were diagnosed with UC, and 8 patients (22.2%) were diagnosed with CD. We found that a proportion of patients who were not reclassified did not receive regular colonoscopies to reconfirm the diagnosis, and this may explain why there were fewer reclassified patients in our study.

The relatively accurate diagnosis of IBDU can be of great use in the selection of surgical procedures like IPAA. Earlier studies have shown that compared with UC patients, IBDU patients with IPAA showed a higher rate of pouch failure and complications [26–29]. Conversely, in recent studies, the long-term functional outcome and pouch failure rate of CD or IBDU patients were identical to those of UC. But there was still a remarkable increase in IBDU or IC patients in rates of pelvic sepsis and fistula [10, 30]. In this present study, 15 IBDU patients (40.5%) underwent surgical intervention, of which 4 patients received IPAA. 2 had postoperative pathology features in line with UC but no postoperative complications were observed. However, 1 patient with a postoperative diagnosis of CD was found with a pouch fistula. This is consistent with several studies that demonstrated that IC patients with pathological features favoring CD had a higher rate of pouch-related complications after IPAA [25, 31]. Therefore, it is imperative to identify predictive markers for CD reclassification. Our study found that the complication of hemorrhage at diagnosis was associated with CD reclassification, while hypoalbuminemia at diagnosis was associated with UC reclassification. Although the sample size was limited, this is one of the first studies to evaluate potential factors about IBDU reclassification. Another study about pediatric-onset IBDU showed that patients with a family history of CD and treatments including surgery, steroids, cyclosporine, anti-TNF and nutritional support were more likely to be diagnosed with CD [32]. Additionally, Sharon Z. Koh et al. discovered that the only clinical factor related to CD reclassification after IPAA for IBDU was younger age at disease onset [33]. As our IBDU patients commonly present for medical evaluation during the intermediate phase, individualized treatment can be implemented based on the identified factors mentioned above before reaching the final differentiation. For IBDU patients who are at risk of transitioning into UC, escalating therapies and IPAA surgery may be considered, while for those at risk of progressing into CD, de-escalation therapies could be contemplated.

Our study described some of the characteristics of IBDU, but this was a single-center retrospective study with a small sample size and a relatively short follow-up period. A multi-center and prospective study is warranted to achieve more clinical data. In addition, approximately one-third of pediatric IBDU patients were reported to display UGI involvement [8]. In the revised Porto Criteria for pediatric IBD-U [23], focally enhanced gastritis, ulceration, or cobblestoning of the stomach are essential features for the identification of IBDU. However, due to 30% of IBDU patients lacking relevant assessment of UGI in this study, only 10% of patients showed UGI involvement, and specific manifestations of the involvement were not further explored.

## Conclusions

In conclusion, we are inclined to recognize IBDU as a distinct disease entity, exhibiting characteristics of both UC and CD. However, distinct from the other two groups, IBDU typically demonstrated a more aggressive onset and a gradual progression over time. Besides, the clinical presentation and therapeutic regimen of IBDU were more similar to those of CD, but it lacked the diagnostic pathological features of CD. The presence of transmural inflammation or crypt abscess, along with the absence of transmural lymphoid aggregates or fissuring ulcers in IBD patients, should highly suggest a diagnosis of IBDU. During follow-up, 24.3% of IBDU patients were re-diagnosed with UC, while 21.6% were re-diagnosed with CD. We observed that the complication of hemorrhage may be an associated factor for CD reclassification and hypoalbuminemia at diagnosis may be an associated factor for UC reclassification. For IBDU patients with concurrent bleeding, a more comprehensive assessment should be conducted before considering IPAA surgery.

## Data Availability

The datasets used and/or analyzed during the current study are available from the corresponding author on reasonable request.

## Abbreviations

5-ASA: 5-Aminosalicylic Acid
CD: Crohn’s disease
IBD: Inflammatory bowel disease
IBDU: Inflammatory bowel disease unclassified
IC: Indeterminate colitis
IFX: Infliximab
IPAA: Ileal pouch-anal anastomosis
PUMCH: Peking Union Medical College Hospital
UC: Ulcerative colitis
UGI: Upper gastrointestinal
